# Transcriptomic Profiling Analysis of Castration-Resistant Prostate Cancer Cell Lines Treated with Chronic Intermittent Hypoxia

**DOI:** 10.3390/cancers14163959

**Published:** 2022-08-16

**Authors:** Chung Lyul Lee, Minji Lee, Ji Yong Lee, Sin-hyoung Hong, Seung Woo Yang, Ji-hyeon Min, Dong-eon Lee, Joonyoung Baek, Chanseul Kim, Jae Sung Lim, Ki Hak Song, Ju Hyun Shin, Gun-Hwa Kim

**Affiliations:** 1 Department of Urology, College of Medicine, Chungnam National University, 266, Munhwa-ro, Jung-gu, Daejeon 35015, Korea; 2 Department of Bio-Analytical Science, University of Science and Technology (UST), Daejeon 34113, Korea; 3 Research Center for Bio convergence Analysis, Korea Basic Science Institute (KBSI), Ochang 28119, Korea; 4 Graduate School of Analytical Science and Technology (GRAST), Chungnam National University, Daejeon 34134, Korea

**Keywords:** castration-resistant prostate cancer, hypoxia, hypoxia-resistant cell lines, RNA-seq

## Abstract

**Simple Summary:**

Prostate cancer is the second most frequently diagnosed cancer and the fifth cause of cancer mortality among men. Although localized and confined tumors are relatively curable, patients with advanced metastatic prostate cancer are still problematic. Hypoxia, which is a marked characteristic of advanced solid tumors, has been suggested to induce the progression of prostate cancer. This study aimed to evaluate the impact of chronic intermittent hypoxia on a castration-resistant prostate cancer cell line in inducing cancer progression using RNA sequencing analysis. Through RNA sequencing analysis, we prove that COL13A1, which is a key factor for the progression of metastasis, is closely related to metastatic prostate cancer. These results suggest that our findings indicate a novel strategy for the clinical management of mCRPC.

**Abstract:**

Castration-resistant prostate cancer (CRPC) is still a major concern in men’s health, with 375,000 cancer deaths annually. Hypoxia, which is a marked characteristic of advanced solid tumors, has been suggested to induce prostate cancer towards CRPC, metastasis and treatment resistance. To evaluate the effect of hypoxia on prostate cancer, two and five cycles of hypoxia and reoxygenation were administered using 22Rv1 cell lines and denominated as 22Rv1-CI and 22Rv1-PCI, respectively. Cancer cell migration was promoted in 22Rv1-CI compared to controls, and the expression of COL13A1 was significantly up-regulated in 22Rv1-CI according to differentially expressed gene analysis of RNA sequencing among groups. Cancer cell migration was impeded in a wound healing assay after transfecting si-COL13A1. Moreover, the expression of COL13A1 was also higher in the cell line originating from bone metastatic prostate cancer compared to other cell lines. Using the open database GEO, we also confirmed that the expression of COL13A1 was higher in bone metastatic prostate cancer tissue than in localized prostate cancer tissue in patients. Therefore, COL13A1 may be closely related to the bony metastasis of prostate cancer, and our findings may provide valuable information on the pathophysiology of the metastatic niche induced by hypoxia in patients with CRPC.

## 1. Introduction

Prostate cancer is the second most frequently diagnosed cancer and the fifth cause of cancer mortality among men, despite numerous ongoing studies. Approximately 1.4 million patients are newly diagnosed and 375,000 deaths occur worldwide in one year [1]. For patients with metastatic castration-resistant prostate cancer (mCRPC), chemotherapeutic agents, such as docetaxel and cabazitaxel, and second-line androgen pathway inhibitors are primarily used in sequence, but they show a survival advantage of only a few months [2].

Hypoxia and angiogenesis are marked characteristics of advanced solid tumors [3]. The efforts of hypoxic cancer cells to survive and adapt under prolonged hypoxia have been suggested to increase tumor invasiveness, treatment resistance and eventually metastasis [4,5,6,7]. In prostate cancer, hypoxia-inducible factor (HIF), a central coordinator of responses to hypoxia, is frequently observed and suggested to affect the progression of prostate cancer [8]. Chronic hypoxia is suggested to induce not only CRPC but also early biochemical relapse, local recurrence and metastasis in patients with localized prostate cancer [9,10,11,12].

The 22Rv1 human prostatic carcinoma cell line is the representative CRPC cell line that expresses androgen receptor splice variant 7 [13,14]. Moreover, the 22Rv1 cell line was derived from a primary prostate cancer specimen in a patient with bone metastasis and showed metastatic potency when grafted into immune-compromised mice [15,16]. Thus, the 22RV1 cell line provides a useful in vitro/in vivo model system for investigating the molecular mechanisms responsible for metastatic progression to CRPC in primary prostate cancer.

In the present study, we applied next-generation RNA sequencing (RNA-seq) technology to discover the transcriptional changes in 22Rv1 cells versus hypoxia-treated 22Rv1 cells [17]. Through analysis of differently expressed genes in each group, we evaluated the types of transcriptional changes in CRPC that occurred under a hypoxic tumor microenvironment (TME) to investigate how they affect the acquisition of aggressiveness and progression in cancer.

## 2. Materials and Methods

### 2.1. Cell Culture

Human prostate carcinoma 22Rv1 cells were obtained from the American Type Culture Collection (Manassas, VA, USA). The 22Rv1 cells were cultured in Roswell Park Memorial Institute (RPMI) 1640 medium supplemented with 10% fetal bovine serum and 100 U/mL penicillin G and 100 μg/mL streptomycin sulfate at 37 °C in a humidified 20% O_2_ and 5% CO_2_ incubator. Media and other cell culture materials were from Invitrogen Corporation (Gaithersburg, MD, USA).

### 2.2. Generation of Hypoxia-Treated 22Rv1 Cell Lines

The effect of hypoxia on cancer cells is subject to the gradient dynamics of oxygen concentration, based on which cellular hypoxia can be mainly classified into acute, chronic and intermittent (cyclic) hypoxia. As the exact categorization of acute and chronic hypoxia in terms of time has not been established, we referred to previous studies and defined the following: chronic hypoxia denotes prolonged periods of low oxygen tension (>24 h), and acute hypoxia denotes a few minutes to a few hours (4~24 h) of oxygen deprivation [18]. To generate chronic intermittent hypoxia-treated cells, the following regimen was used [19]: control cells were cultured in normal oxygen conditions; chronic intermittent hypoxia-treated cells (denominated as 22Rv1-CI) were cultured by periodic exposure to cycles of hypoxia (1% O_2_ for 24 h) and reoxygenation (5% CO_2_ referred as 20% O_2_ for 24 h) (H–R cycles) and two H–R cycles were repeated; prolonged chronic intermittent hypoxia-treated cells (denominated as 22Rv1-PCI) were treated with five H–R cycles. Under hypoxic stress, cells were placed into a modular incubator chamber that was purged with a 1% O_2_ and 99% N_2_ gas mixture for 5 min (flow rate of 20 L/rain). This chamber was then placed into an incubator maintained at 37 °C for 24 h. We then carefully separated the viable cells from treated cells and sub-cultured them under normal oxygen conditions. 

### 2.3. Cell Viability Assay

To assess cell viability and resistance against hypoxic treatment, the treated and control cell lines (2 × 105 cells/well) were seeded onto six-well plates for 24 h and then treated with 1% O_2_ and 99% N_2_ gas for 1–5 days. After the treatment, the ratio of cytotoxicity was assessed using the MTT assay. The cells were treated with 0.5 mg/mL of MTT reagent (3- (4,5-dimethylthiazol2-yl)-2,5-diphenyltetrazolium bromide) for 1 h, after which precipitated formazan crystals were dissolved using dimethyl sulfoxide, and the optical density was measured at 450 nm using a spectrophotometer. 

### 2.4. Western Blotting

The 22Rv1 cells and the established hypoxia-treated cells were treated using Pro-Prep Protein Extraction Solution (iNtRON, Seongnam, Korea). After centrifugation, protein concentrations in supernatants were determined using Micro BCA protein assay kits (Pierce Chemical, Dallas, TX, USA), with bovine serum albumin being used as a standard. Aliquots containing 20 μg protein were analyzed by 12% sodium dodecyl sulfate polyacrylamide gel electrophoresis and transferred to PVDF membranes. For immunoblotting, membranes were incubated in 5% skim milk in 0.3% TritonX-100 in PBS (PBST) for 1 h and incubated overnight at 4 °C with the HIF-1α primary antibodies. The membranes were washed three times for 10 min each in PBST, followed by incubation for 1 h with peroxidase-labeled secondary antibodies. After three more washes, immune-labeled proteins were detected by using a SuperSignal enhanced chemiluminescence kit (Pierce Chemical) and BioMax Light-1 film (Eastman Kodak, Rochester, NY, USA), and quantitated using a Western blot detection system (Bio-Rad, Hercules, CA, USA), with the density of each band normalized to that of GAPDH.

### 2.5. RNA-Seq Analysis

Total RNA was extracted by the RNeasy Plus Mini Kit (Qiagen, Valencia, CA, USA, following the manufacturer’s instructions. RNA quality was evaluated by analysis of the RNA integrity number determined using a 2100 bioanalyzer (Agilent RNA 6000 Nano Kit, Agilent Technologies, Santa Clara, CA, USA). Library preparation was performed using Advanta RNA-seq XT NGS Library Prep Kits for Fluidigm JUNO. Sequencing was carried out on an Illumina NextSeq 550. The raw FASTQ files were filtered by Trimmomatic. Filtered FASTQ files were mapped and counted with STAR 2.7, and differential gene expression analyses of control and hypoxia-treated samples were performed using the DESeq2 [20]. Significant genes were identified by using the cut-off values of *p*-value < 0.05 and fold change (FC) in mean expression of |FC| > 1.5. Volcano plots of differentially expressed genes (DEGs) were generated using R packages. 

### 2.6. Gene Set Enrichment Analysis (GSEA)

GSEA was performed to investigate the rank genes according to expression variation between 22Rv1 and chronic intermittent hypoxia-treated cells in the enrichment of the MSigDB Collection [21,22].

### 2.7. Ingenuity Pathway Analysis (IPA)

The list of DEGs was uploaded into QIAGEN’s IPA “QIAGEN Ingenuity Pathway Analysis (IPA). Available online: http://www.ingenuity.com (accessed on 8 May 2022)”. Biological networks and pathways were analyzed to study the direct or predicted relationships between control and chronic intermittent hypoxia-treated samples.

### 2.8. Acquisition and Filtering DEG of Dataset from the Gene Expression Omnibus (GEO) Database

We identified suitable datasets of patients with prostate cancer in the “Gene Expression Omnibus (GEO). Available online: https://www.ncbi.nlm.nih.gov/geo/ (accessed on 27 May 2022)” (database, which is a public database of gene expression profiles and sequence-based data that is freely available for users. The size of datasets and the unity of the platform were evaluated, and a gene expression profile dataset (GSE32269) was selected and downloaded from GEO. Expression profiling of GSE32269 was prepared by array biotinylated cRNA according to the standard Affymetrix protocol. GSE32269 contained 51 patients with prostate cancer, 22 of whom had primary prostate cancer, while 29 patients had a metastatic bone lesion of prostate cancer [23]. Datasets were analyzed using the limma R package, applied to filter the differently expressed genes (DEGs) between the groups of patients with primary and metastatic prostate cancer. Significant DEGs were selected at values |log2fold change| > 2 and *p*-value < 0.05.

### 2.9. RNA Extraction and Quantitative Polymerase Chain Reaction (PCR) Analysis

Total RNA was extracted from prostate cancer cell lines and we established chronic intermittent hypoxia-treated cells by adding tissue samples to 1 mL Trizol (Invitrogen, Waltham, CA, USA) and cell harvesting. The cell lysates were transferred to 1 mL tubes, mixed with 200 μL chloroform, and incubated for 5 min at room temperature. The cell lysates were centrifuged at room temperature for 10 min at 13,000 rpm, and the supernatants were transferred to clean tubes containing 1 mL isopropyl alcohol (Sigma-Aldrich, St. Louis, MO, USA), followed by centrifugation for 30 min at 13,000 rpm. Each resulting supernatant was mixed with 1 mL DEPC-treated water (Sigma-Aldrich) and centrifuged for 10 min at 13,000 rpm. These supernatants were discarded, and the pellets were dried at room temperature, melted into DEPC-treated water (Sigma-Aldrich), and preserved at −75 °C. The integrity and quality of RNA were verified by agarose gel electrophoresis. One-microgram aliquots of RNA were reverse-transcribed to cDNA using random primers and a First-Strand cDNA Synthesis Kit (Enzynomics, Daejeon, Korea), with incubations at 42 °C for 5 min, 50 °C for 60 min, and 95 °C for 5 min. Sequences of interest were amplified from cDNA by PCR. The amplification consisted of an initial denaturation for 5 min at 95 °C, followed by 35 cycles of denaturation at 95 °C for 30 s; annealing at 58 °C for 30 s; extension at 72 °C for 30 s; and a final extension at 72 °C for 5 min. PCR products were analyzed by electrophoresis on 1.2% agarose gels and normalized relative to GAPDH mRNA in the same samples.

### 2.10. Small Interfering RNA (si-RNA) and Transfections

All si-RNAs were purchased from Bioneer (Daejeon, Korea). Transient transfection of each si-RNA for COL13A1 knockdown was conducted using Lipofectamine^®^ 2000 transfection reagent (Invitrogen; Thermo Fisher Scientific, Inc., Waltham, MA, USA), according to the manufacturer’s protocols. The cells were transfected with COL13A1 or (NC)-si-RNA (si-NC) for 48 h at a final concentration of 25 pmol, and the efficiency of each si-RNA oligo duplex was confirmed by reverse transcription (RT)-PCR.

### 2.11. Wound Healing Assay

The treated cells and controls were seeded in a 12-well plate at a concentration of 1 × 10^5^ cells /well. After attaining 80% of confluence, the cell cultures were scratched with a 200 μL sterile pipette tip using the designed mold. Then, the detached cells were washed away with PBS (1X). Subsequently, 1 mL of RPMI conditioned medium was added. Vertical reference lines on the bottom of the plate were made with an ultrafine tip marker to have a grid for alignment to obtain the same field for each image acquisition run. Once the reference lines were made (approximately 3000 μm of distance), the plate was placed under a phase-contrast microscope. We analyzed selected regions of interest using a Zeiss inverted microscope on a 4X objective with NA/0.25 every 24 h for 48 h. The scratch area was measured using ImageJ software (National Institutes of Health, Bethesda, MD, USA). Migration rate was expressed as the percentage of scratch closure on an initial area basis, according to the following equation:

Wound course% = {(A_t=0_ − A_t=Δt_)/(A_t=0_)} × 100 (1)

where A_t=0_ is the initial wound area; A_t=Δt_ is the wound area after 24 h and 48 h of the initial scratch.

### 2.12. Statistical Analysis

Data were visualized by bar charts or box plots, and the Student’s *t*-test, Mann–Whitney U-test, or Kruskal–Wallis test was applied for statistical analysis, as appropriate. PRISM software version 8.02 (San Diego, CA, USA) was used for statistical analyses and plotting the data. *p* < 0.05 was considered statistically significant.

## 3. Results

### 3.1. Generation of Hypoxia-Resistant 22Rv1 Cell Lines

**Figure 1:**
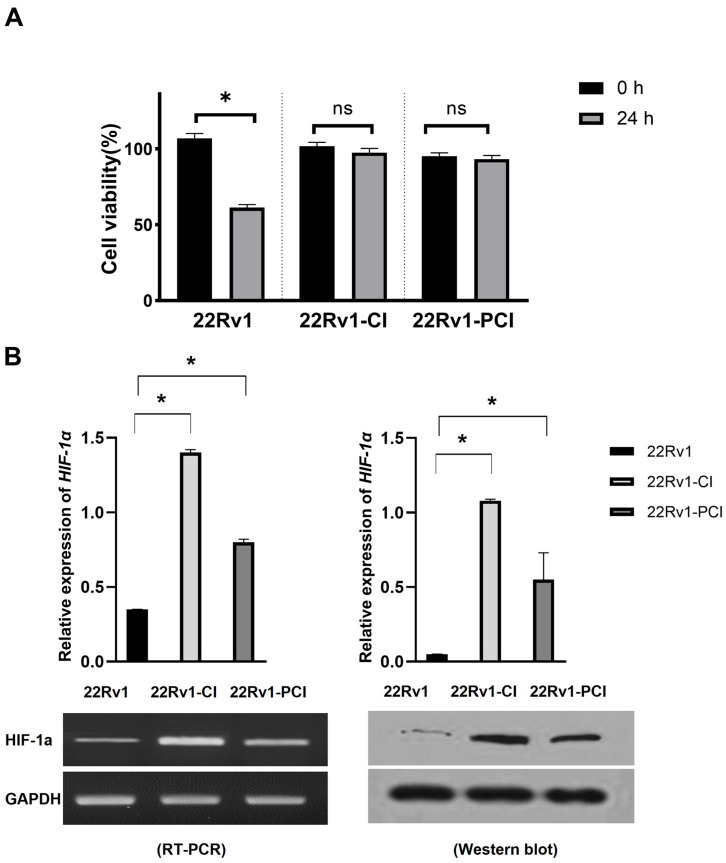
Characterization of hypoxia-resistant cell lines. (**A**) Identification of cell viability in hypoxic environment using MTT assay in 22Rv1 cells or hypoxia-resistant cells (22Rv1-CI and 22Rv1-PCI cell lines). (**B**) mRNA expression and Western blot for HIF-1α expression in hypoxia-resistant cells (22Rv1-CI and 22Rv1-PCI cell lines) compared with 22Rv1 cells. Data are mean ± SEM. * *p* < 0.05; ns, not significant.

Hypoxia is detrimental to normal as well as cancer cells. Cancer cells can adapt to hypoxia by altering the expression of genes related to the metabolic process, proliferation, and angiogenesis [24]. Chronic hypoxia is a persistent reduction in oxygen and results in cancer cell death. However, cycling hypoxia/reoxygenation stress changes the TME to adapt to the situation [22,25]. Cancer progression (angiogenesis, metastasis, treatment resistance, and stemness) is more associated with intermittent hypoxia than with acute or prolonged chronic hypoxia [18,19,26]. In this study, we inflicted chronic intermittent hypoxia treatment on 22Rv1 cells; 22Rv1-CI and 22Rv1-PCI cells presented more resistance to hypoxia, as indicated in the MTT assays (Figure 1A). We validated whether the cells had been properly induced with hypoxia by performing Western blotting for HIF-1α (Figure 1B and Figure S1). 22Rv1-CI cells showed more HIF-1α expression in Western blot and RT-PCR than 22Rv1-PCI.

### 3.2. Comparative Analysis of the Transcriptomic Profiling of Hypoxia-Resistant Cells

**Figure 2:**
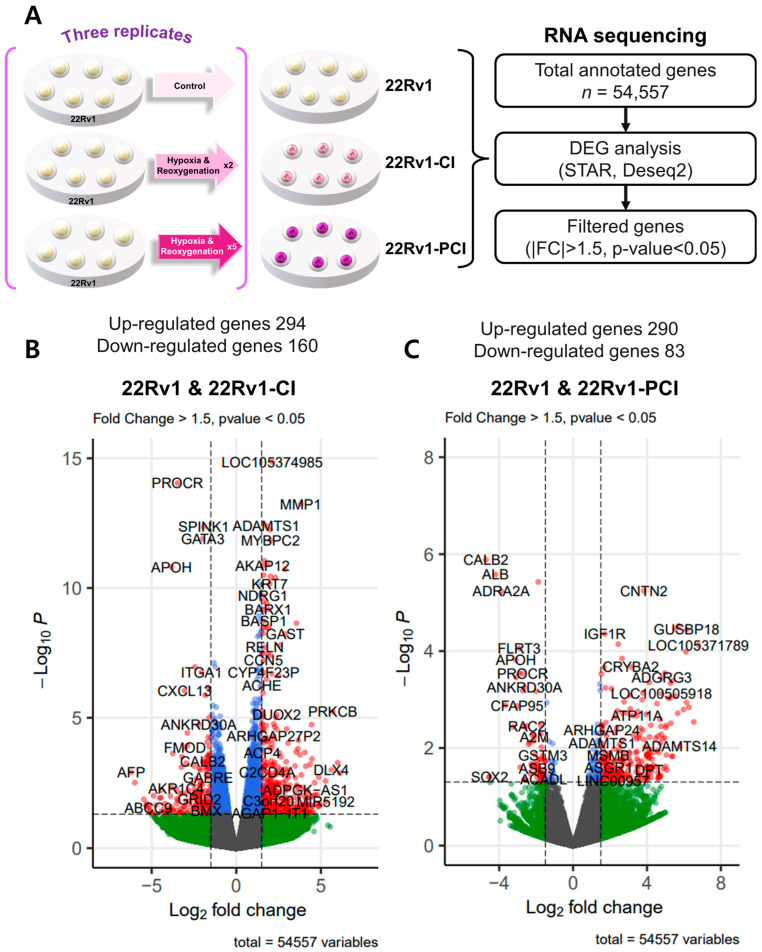

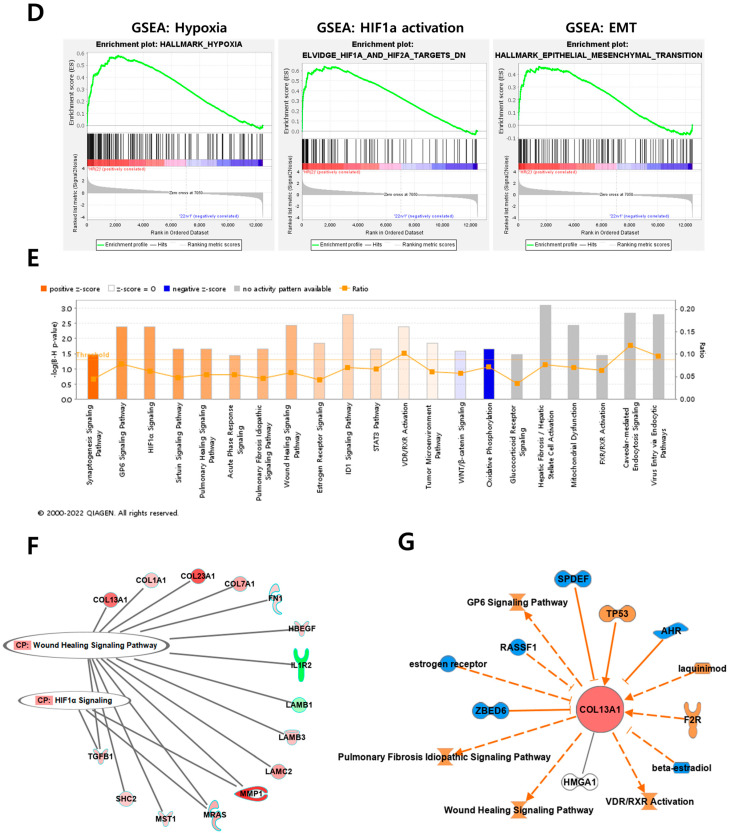

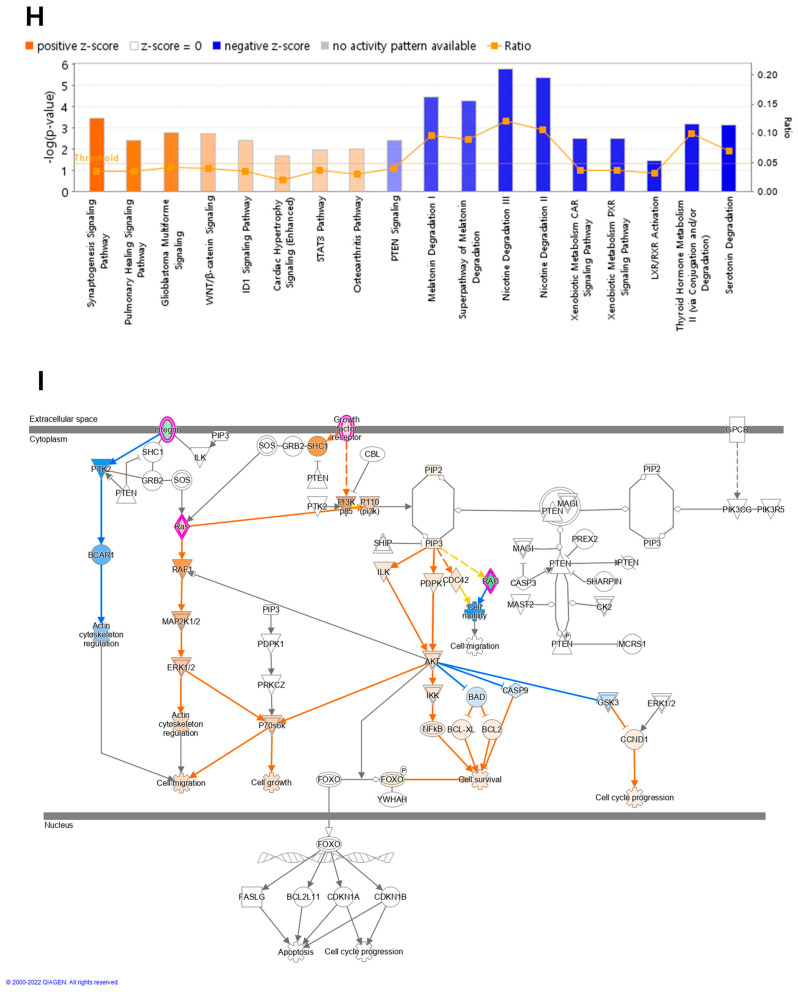

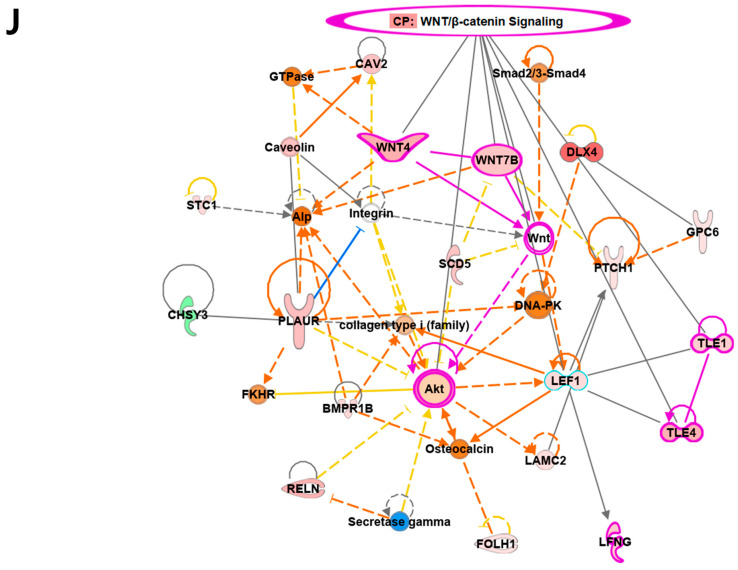
Overview of transcriptome profiles between 22Rv1 cells and hypoxia-resistant cells. (**A**) Schematic outline of RNA sequencing in 22Rv1 cells or hypoxia-resistant cells. (**B**,**C**) Volcano plot in 22rv1-CI and 22rv1-PCI displaying fold change versus adjusted *p*-values. Red dots represent the significant DEGs (|FC| > 1.5, *p*-value < 0.05). (**D**) GSEA enrichment score curve of hypoxia- or migration-related genes in 22rv1-CI. (**E**) Top significantly affected canonical pathway analysis in 22rv1-CI. (**F**) Gene network analysis associated with hypoxia and wound healing signaling in 22rv1-CI. Red, upregulated; green, downregulated. (**G**) Representative gene networks of Col13A1 in 22rv1-CI. (**H**) Top significantly affected canonical pathway analysis in 22rv1-PCI. (**I**) PTEN canonical pathway in 22rv1-PCI. (**J**) Wnt pathway in 22rv1-PCI.

To investigate differently expressed genes (DEGs), we performed transcriptomic RNA sequencing in 22Rv1 and two different hypoxia-resistant cell lines. Through RNA-seq analysis, we identified 454 differently expressed genes (294 upregulated and 160 downregulated) in 22rv1-CI cells and 373 differently expressed genes (290 upregulated and 83 downregulated) in 22rv1-PCI cells (Tables S1 and S2). Volcano plots were generated to visualize the distribution of DEGs between 22Rv1 cells and cells in each treated group, with |FC| > 1.5 and *p*-value < 0.05 (Figure 2A–C).

To identify the potential mechanism of 22Rv1-CI cells, Gene Set Enrichment Analysis (GSEA) was performed. GSEA analysis showed that several genes of 22Rv1-CI were positively correlated with hypoxia and HIF-α activation (Figure 2D). In particular, HIF-1α was significantly increased in 22Rv1-CI compared with 22Rv1 cells, while it was slightly decreased in 22Rv1-PCI (Figure 1A). Moreover, epithelial-to-mesenchymal transition (EMT)-related genes, which induce migration or invasion for metastasis in cancer cells, were highly enriched in 22Rv1-CI. From these results, we can infer that chronic intermittent hypoxia may induce the HIF signaling pathway in 22Rv1 cells, with different subtypes of HIF-α as more H-R cycles are repeated. Next, further study of the molecular network and pathway analysis were performed using Ingenuity Pathway Analysis (IPA) in hypoxia-resistant cells. In the 22Rv1-CI cells, top canonical pathways included significant enrichment in the HIF1-α pathway and wound healing pathway (Figure 2E). Moreover, we found highly expressed genes (COL13A1, COL23A1, MMP1, TGFB1) involved in these pathways (Figure 2F). We reviewed the results of DEG analysis of 22Rv1 and 22Rv1-CI cells related to cell adhesion and migration; the expression of the COL family was found to be significantly upregulated. Further, IPA was reversely carried out, and COL13A1 was revealed to be directly and indirectly related to cell adhesion and wound healing pathways (Figure 2G). To determine the reason that 22Rv1-PCI cells showed quite different patterns compared with 22Rv1-CI, molecular and pathway analyses were performed using IPA. Through top canonical pathway analysis, we found that prolonged chronic intermittent hypoxia-resistant cell lines were associated with cell proliferation through the PTEN signaling pathway and Wnt signaling pathway (Figure 2H–J). PTEN, which is a tumor suppressor, regulates the PI3K/AKT/mTOR pathway involved in cell survival and proliferation. Moreover, 22Rv1-PCI related genes are negatively correlated with the PTEN signaling pathway, promoting cell growth and cell cycle progression. Wnt signaling is also related to tumor progression and metastasis. Activation of the Wnt signaling pathway promotes cell proliferation and migration in prostate cancer. Together, the 22Rv1-PCI cells partly correlated with cell migration but they are predicted to focus more on cell proliferation and cell growth.

### 3.3. Cancer Cell Migration Is Promoted in the Survival Cells from Chronic Intermittent Hypoxia Treatment, but Prolonged Hypoxia Impedes Migration

**Figure 3:**
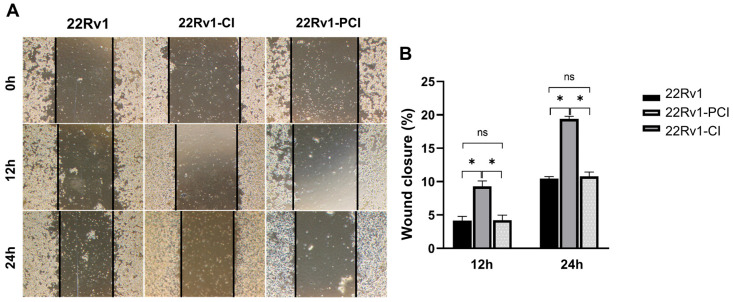
(**A**) Cell migration of 22Rv1-CI or 22Rv1-PCI cells compared with 22Rv1 cells. (**B**) Bar graph represents the percentage of scratch closure. Data are mean ± SEM. * *p* < 0.05; ns, not significant.

As mentioned above, hypoxic environments induce increased tumor invasiveness, metastasis, and treatment resistance. HIF-1α is a key factor for cancer cells to adapt and survive [7,27]. Furthermore, GSEA and canonical pathway analysis proved that the tendency for cell adhesion and migration is significantly upregulated in hypoxia-resistant cells. We performed wound healing assays to evaluate whether hypoxia had induced cell migration in the survival cells. Here, 22Rv1-CI remarkably enhanced cell migration over control and 22Rv1-PCI cells (Figure 3A,B). Moreover, there was no statistical difference in the cell migration of 22Rv1 and 22Rv1-PCI cell lines. These results indicate that as cancer cells undergo chronic intermittent hypoxia, genes or signaling pathways related to cell migration are induced first; however, as chronic intermittent hypoxia is prolonged, there would be some points where different signaling pathways and RNAs are induced.

### 3.4. Chronic Intermittent Hypoxia Upregulates COL Families including COL13A1

**Figure 4:**
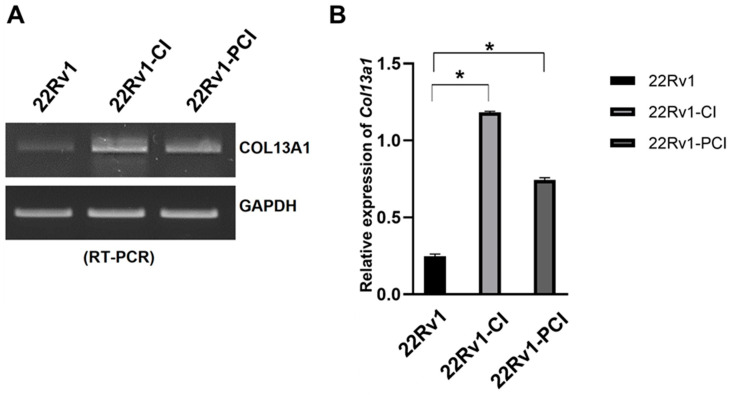
mRNA expression of COL13A1 22Rv1-CI, and 22Rv1-PCI cells compared with 22Rv1 cells. (**A**) mRNA expression of COL13A1 in 22Rv1 cells or cells with repeated H–R cycles. (**B**) Bar graph represents the expression of COL13A1. Data are mean ± SEM. * *p* < 0.05.

The simultaneous collagen deposition and degradation under hypoxia, which is induced by HIFs and affects cell adhesion and migration, is a marked characteristic of a cancer cell [28]. Therefore, we focused on COL families from DEGs of RNA-seq. The expression of four different COL genes (COL13A1, COL1A1, COL23A1, and COL7A1) was significantly upregulated in 22Rv1-CI compared to 22Rv1 cells (Table 1). However, none of the COL genes were significantly upregulated in 22Rv1-PCI compared to 22Rv1 cells; rather, COL18A1, COL13A1, and COL6A1 were downregulated in 22Rv1-PCI compared to 22Rv1-CI (Table S3). In the wound healing assay, cell migration was initially increased (22Rv1-CI) but decreased in prolonged chronic intermittent hypoxia (22Rv1-PCI). With the results of wound healing assay, because the expression of collagens was upregulated in 22Rv1-CI and downregulated in 22Rv1-PCI, we selected collagens as target genes that may affect cell migration. Of these collagens, we chose COL13A1 based on IPA network and pathway analysis. We confirmed the expression of COL13A1 in each group by RT-PCR and found the same pattern of up- and downregulation of COL13A1 expression as more H–R cycles were repeated (Figure 4 and Figure S2).

### 3.5. COL13A1 Silencing Inhibits Prostate Cancer Cell Migration Regardless of Cell Viability

**Figure 5:**
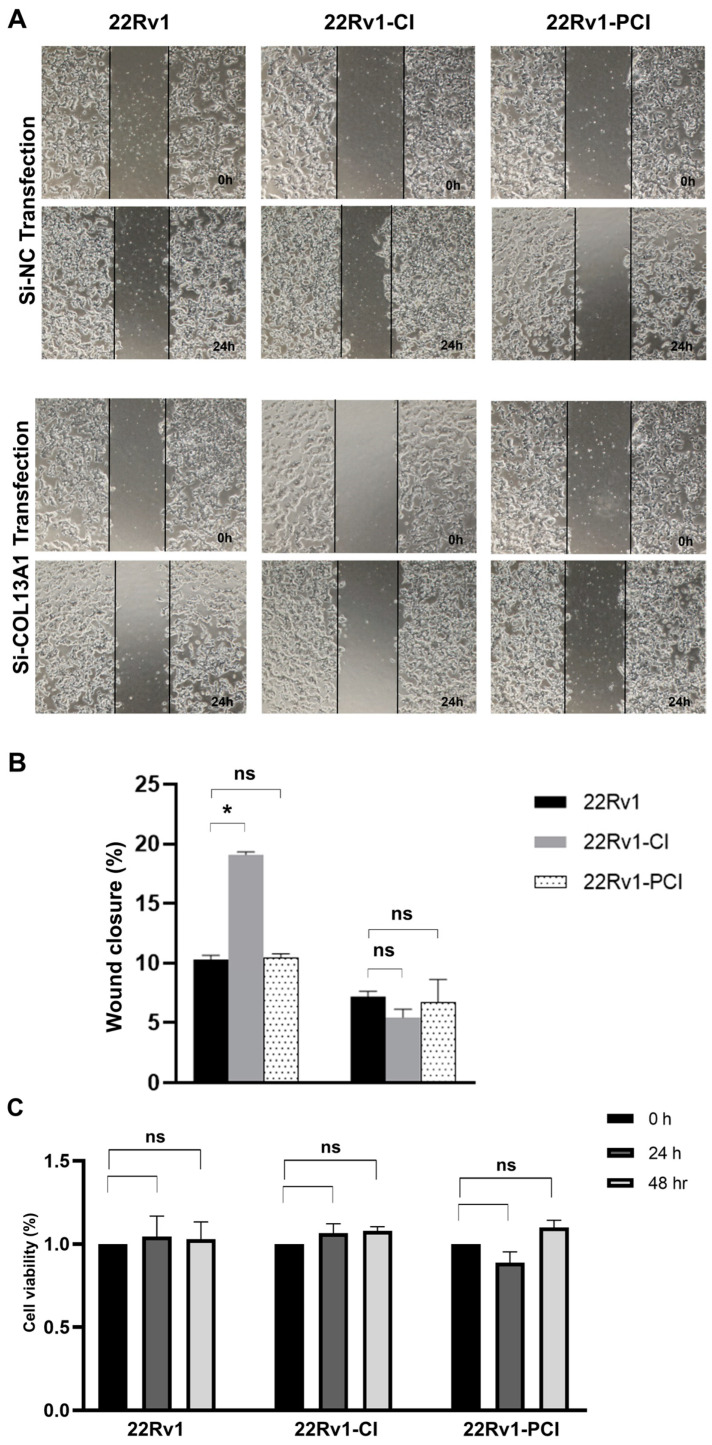
Wound healing assay of 22Rv1, 22Rv1-CI, and 22Rv1-PCI cells transfected with si-NC or si-COL13A1. (**A**) Representative image of wound healing assay in control and hypoxia-resistant cells. (**B**) Bar graph represents the percentage of scratch closure. (**C**) Cell viability assay of 22Rv1, 22Rv1-CI, and 22Rv1-PCI cells transfected with si-NC and si-COL13A1. Data are mean ± SEM. * *p* < 0.05; ns, not significant.

To further evaluate whether COL13A1 is associated with cancer cell migration, we firstly confirmed the expression of COL13A1 by PCR. Expression of COL13A1 was then transiently silenced by si-COL13A1 in 22Rv1-CI, 22Rv1-PCI, and control cells, respectively, with si-NC being used as a control. The ability of cells to migrate was assessed by wound healing assays. Downregulated expression of COL13A1 by si-RNA markedly impeded cell migration ability compared with 22Rv1 or 22rv11-PCI (Figure 5A,B). Additionally, to further understand the effect of COL13A1 on cancer cell viability, an MTT assay was performed after si-COL13A1 transfection to each group for 24 h and 48 h. As a result, transfection of si-COL13A1 showed no effect on cell viability, regardless of chronic intermittent hypoxia treatment (Figure 5C).

### 3.6. Expression of COL13A1 Is Especially Increased in PC3 Cells

**Figure 6:**
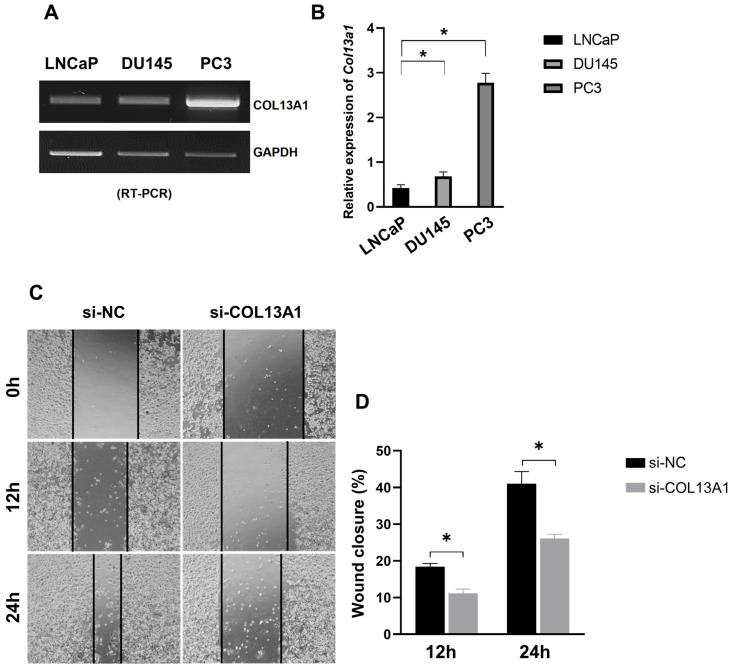
Expression of COL13A1 in LNCaP, DU145, and PC3 prostate cancer cell lines. (**A**) mRNA expression of COL13A1 in three differently originated prostate cancer cell lines. (**B**) Bar graph of the relative expression of COL13A1 in several prostate cancer cell lines. (**C**) Representative image of wound healing assay in control and hypoxia-resistant cells. (**D**) Bar graph represents the percentage of scratch closure. Data are mean ± SEM. * *p* < 0.05.

To understand whether the expression of COL13A1 is related to the metastasis of prostate cancer, we evaluated the expression of COL13A1 in three differently originated prostate cancer cell lines, namely LNCaP (hormone-sensitive, from the lymph node), DU145 (castration-resistant, from the brain), and PC3 (castration-resistant, from the bone). In PC3 cell lines (Figure 6A,B and Figure S3), the expression of COL13A1 was significantly increased compared to other cell lines. Additionally, we further confirmed the effect of COL13A1 on the migration capability of PC3 cell lines. We showed that the ability of cell migration was significantly reduced in PC3 cells treated with si-RNA transfection of COL13A1 compared to si-NC in a time-dependent manner (Figure 6C,D). Therefore, COL13A1 was highly correlated with the metastasis of prostate cancer.

### 3.7. Expression COL13A1 Is Increased in Metastatic Bone Lesions of Human Prostate Cancer

**Figure 7:**
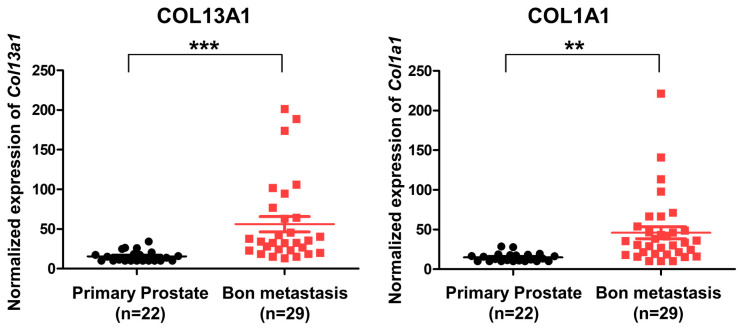
Expression of COL13A1 and COL6A1 in patients with primary localized prostate cancer and bone metastatic prostate cancer (GSE32269). The expression of COL13A1 and COL6A1 was high in DEGs of bone metastatic prostate cancer (cut-off value: log2FC > 2, *p*-value < 0.05). Data are mean ± SEM. ** *p* < 0.01, *** *p* < 0.001.

To evaluate the expression of COL13A1 and confirm that it is associated with metastatic bone lesions in human prostate cancer, we used an open GEO database. Using the published gene set of GSE32269, microarray data of 22 patients with primary prostate cancer and 29 patients with metastatic bone lesions of prostate cancer were analyzed [23]. Our results showed that the expression of different types of collagen subfamilies was increased, including COL13A1 and COL1A1 (Figure 7). Since the expression of COL13A1 was increased in PC3 cell lines that were derived from bone metastatic prostate cancer in RT-PCR, we can infer that COL13A1 may be closely related to the bony metastasis of prostate cancer.

## 4. Discussion

Solid tumors necessarily encounter temporary, progressive, intermittent, or profound hypoxia because of insufficient blood supply [29]. The dynamic gradient of O2 diffusion and consumption is detected in the TME with different regions and degrees of hypoxia [24]. The influence of hypoxia and HIF activation on the TME promotes changes in its components, such as the presence of deregulated extracellular matrix (ECM) deposition, cancer-associated fibroblasts, and expanded vascularization with HIF-driven transcriptional responses [30]. Prostate cancer is the representative malignancy in which hypoxia plays a key role in cancer progression [7]. Adaptation to hypoxic stress in prostate cancer is associated with poor clinical prognosis, resistance to chemotherapy and radiotherapy, and increased potency for distant metastasis [9,31,32]. Many studies performed to discover how hypoxia affects the progression and aggressiveness of cancer have focused on intermittent cycling hypoxic stress [19,26,33]. In our study, 22Rv1 cell lines and castration-resistant cells with metastatic potential were treated with chronic intermittent hypoxia by scraping and sub-culturing a few near-death cells. By this method, we generated prostate cancer cell lines with hypoxia resistance, increased cell migration, and differential gene expression, which may represent mCRPC initiating invasion and metastasis. The present study may provide transcriptional insights into a pre-metastatic niche in prostate cancer for future associated studies.

The hypoxic microenvironment of tumors promotes cancer cell migration via interaction between the ECM and cancer cells, mediated by HIF [30]. Collagens, the major components of the ECM that self-assemble into cross-striated fibrils by post-transcriptional modifications, provide not only support for cell growth but also are responsible for the mechanical wound healing process [34]. Under the hypoxic TME, collagen fibrils become stiff and aligned in the ECM, mediated by HIF-1, which results in an increase in the capacity for invasion and migration in cancer [30,35,36]. In our study, the exposure of 22Rv1 cells to hypoxic stress resulted in the promotion of cancer cell migration. Furthermore, the changes in the expression of genes relevant to cell migration and wound healing signaling pathways were revealed by DEG analysis of RNA-seq. GSEA showed that the expression of genes related to hypoxia, HIF-α, and epithelial-to-mesenchymal transition (EMT) was positively correlated in 22Rv1-CI. Accordingly, we focused on differentially expressed collagen genes from DEG analysis, and the expression of COL13A1, COL23A1, COL1A1, and COL7A1 was found to be significantly upregulated in 22Rv1-CI cells. In a recent study, upregulated COL7A1 expression was suggested to have prognostic significance and cause distant metastasis in gastric cancer [37]. COL23A1 is suggested to be highly expressed in metastatic prostate cancer compared with benign prostate tissue [38]. COL1A1 is also known as a key factor in predicting the prognosis and progression of prostate cancer [39]. Taken together, COL families may play important roles in cancer progression induced by HIF under a hypoxic TME.

Among COL families, the expression of COL13A1 was significantly upregulated in 22Rv1-CI cells. Similarly, increased cancer cell migration was detected in the wound healing assay. In contrast, significantly decreased migration was seen after si-COL13A1 transfection. Collagen XIII is a transmembrane protein, and its ectodomain can bind to many proteins, including the α1-subunit of integrin. Because of its location and its binding properties, it has been postulated that collagen XIII is involved in cellular adhesion and migration [40]. Recently, integrin α11β1 was revealed to be a receptor for collagen XIII, and it mediates cell adhesion, which has a role in the regulation of bone homeostasis. Moreover, overexpression of COL13A1 showed significant bone overgrowth, followed by severe osteoporosis, in a mouse model [41]. In previous studies of bladder cancer, silencing of COL13A1 changed the invasion patterns of infiltration and decreased invasion capability through decreased invadopodium. It is also suggested that COL13A1 expression in voided urine can be used as a prognostic factor for bladder cancer [42,43]. In the present study, we detected the increased expression of COL13A1 with an increased cell migration ability after chronic intermittent hypoxic treatment of 22Rv1 cells. Moreover, PC3 cell lines that are derived from bone metastatic tissue of prostate cancer showed increased expression of COL13A1 compared to LNCap and DU145 prostate cancer cell lines; we also confirmed the increased expression of COL13A1 in prostate cancer patients with bony metastasis using the GEO database. Thus, COL13A1 induced by hypoxic stress may play a role in cell migration, adhesion, and infiltration to bones, the most common site where metastases occur in advanced prostate cancer.

In our study, the expression of COL13A1 was significantly upregulated; hypoxia, HIF-1a activation, and EMT were positively correlated, and cell migration was promoted in 22Rv1-CI cells. However, in 22Rv1-PCI cells, with additional cycles of hypoxia, the expression of collagens was downregulated, and cell migration decreased compared to 22Rv1-CI cells. Furthermore, the expression of HIF-1α and COL13A1 in 22Rv1-PCI was suppressed to the level of 22Rv1. Comparing prolonged chronic hypoxia with intermittent hypoxia in vitro, intermittent hypoxia was suggested to endow cancer cells with greater metastatic potential than chronic hypoxia [19]. According to a recent study, the expression of HIF-2α and HIF-3α occurs in a considerably later phase of chronic hypoxia than HIF-1α [44]. Thus, the conversion of HIF-1α to other HIFs, which are more activated in the late phase of prolonged hypoxia, would have negative effects on cell migration and COL gene expression.

Meanwhile, our results showed that the gene expression of 22Rv1-PCI cells was significantly related to PTEN/PI3K/AKT and activation of the Wnt signaling pathway. In recent research, HIFs activated cell survival-related genes in lung cancer with a hypoxic environment. HIF2-α induces the upregulation of PI3K/AKT and promotes the activation of the Wnt signaling pathway through the induction of β-catenin expression. Activation of the Wnt signaling pathway would be important for resistance to hypoxic stress. In addition, the PTEN signaling pathway is negatively correlated with PI3K/AKT signaling. In our results, RAS were highly expressed in the PTEN signaling pathway. RAS promotes lipid secondary messenger phosphatidylinositol 3,4,5-trisphosphate (PIP3) from phosphatidylinositol 4,5-bisphosphate (PIP2) in PI3K/AKT signaling to regulate cell growth and proliferation [45]. Moreover, cell survival- and cell cycle progress-related genes such as PI3K are predicted to increase in 22Rv1-PCI cells. The activation of the PI3K cascade is significantly prevalent in malignancies. Although further study of the prolonged intermittent hypoxic environment is needed, we suggest that prolonged intermittent hypoxic cells are focused on cell proliferation and cell growth resistant to hypoxic stress.

## 5. Conclusions

In this study, we generated novel hypoxia-resistant CRPC cell lines using 22Rv1 cells, which are known to have metastatic potential. Through analyzing the transcriptomic profiling of DEGs, we found COL13A1 upregulated in hypoxia-treated prostate cancer cells. By silencing COL13A1 activity with si-RNA, we discovered that the migration of cancer cells significantly decreases. In addition, we confirmed the effect of COL13A1 in PC3 cell lines, which were derived from bone metastatic prostate cancer. The expression of COL13A1 was significantly increased in PC3 cells and the migration of PC3 cells was highly reduced by silencing COL13A1 activity. Furthermore, using the open database GEO, we also confirmed that the expression of COL13A1 was higher in bone metastatic prostate cancer tissue than in localized prostate cancer tissue in patients.

Therefore, COL13A1 may be closely related to the bony metastasis of prostate cancer, and our findings may provide valuable information on the pathophysiology of the metastatic niche induced by hypoxia in patients with CRPC. These observations have implications for the development of novel strategies for the clinical management of mCRPC in the future.

## Figures and Tables

**Table 1:** DEG analysis comparing COL families among 22Rv1 and chronic intermittent hypoxia-treated cells (22Rv1-CI).

Gene Symbol	log2FoldChange	*p*-Value
COL23A1	2.97750599	2.27 × 10^−5^
COL13A1	2.50566753	2.4 × 10^−7^
COL7A1	1.60152417	8.16 × 10^−6^
COL1A1	1.05868708	0.001011

## Data Availability

Data are contained within this article. Data are not publicly available due to privacy restrictions.

## Supplementary Materials

The following supporting information can be downloaded at: https://www.mdpi.com/article/10.3390/cancers14163959/s1, Figure S1: Uncropped Figure 1B, Figure S2: Uncropped Figure 4A, Figure S3: Uncropped Figure 6A, Table S1: Differently expressed genes in 22Rv1-CI, Table S2. Differently expressed genes in 22Rv1-PCI, Table S3. Differently expressed genes between 22Rv1-CI and 22Rv1-PCI.
