# Supplementary material for: Transcriptomic Profiling Analysis of Castration-Resistant Prostate Cancer Cell Lines Treated with Chronic Intermittent Hypoxia
Source: Cancers (Basel). 2022 Aug 16;14(16):3959. doi: 10.3390/cancers14163959 (PMC9406188; doi:10.3390/cancers14163959)
Supplement: Supplementary file 1 [file cancers-14-03959-s001.zip › Supplementary Materials.pdf]

Supplementary Materials

Figure S1. Uncropped western blot for HIF-1α expression in three different cell lines; 22Rv1, 22Rv1-CI, 22Rv1-PCI.

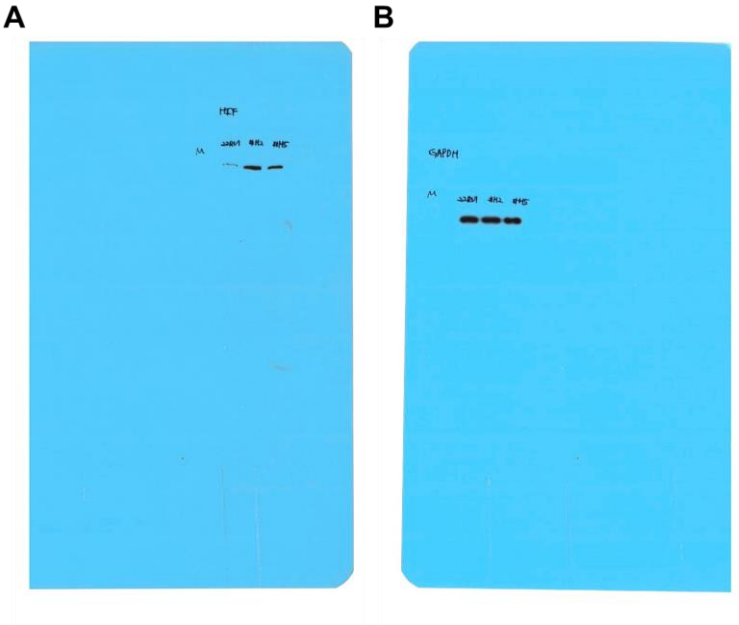

Figure S1. Uncropped western blot for HIF-1α expression in three different cell lines; 22Rv1, 22Rv1-CI, 22Rv1-PCI. (A) Uncropped western blot for HIF-1α. (B) Uncropped western blot for GAPDH.

Figure S2. Uncropped PCR for COL13A1 expression in three different cell lines; 22Rv1, 22Rv1-CI, 22Rv1-PCI.

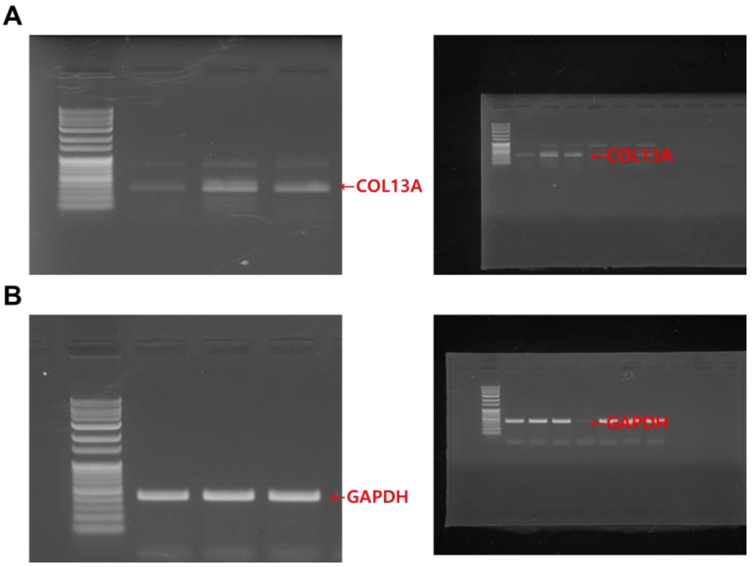

Figure S2. Uncropped western blot for HIF-1α expression in three different cell lines; 22Rv1, 22Rv1-CI, 22Rv1-PCI. (A) Uncropped PCR for COL13A1. (B) Uncropped PCR for GAPDH.

Figure S3. Uncropped PCR for COL13A1 expression in three different cell lines; LNCaP, DU145 and PC3 prostate cancer cell lines.

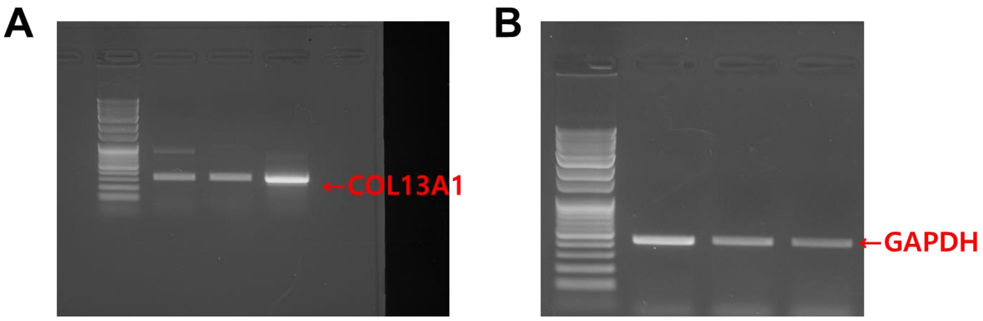

Figure S3. Uncropped PCR for COL13A1 expression in three different cell lines; LNCaP, DU145 and PC3 prostate cancer cell lines. (A) Uncropped PCR for COL13A1. (B) Uncropped PCR for GAPDH.
